# Repair of Retrorsine-Induced DNA Damage in Rat Livers: Insights Gained from Transcriptomic and Proteomic Studies

**DOI:** 10.3390/toxins16120538

**Published:** 2024-12-13

**Authors:** Yun Long, Yiwei Wang, Zijing Song, Xin He, Yisheng He, Ge Lin

**Affiliations:** 1 School of Biomedical Sciences, Faculty of Medicine, The Chinese University of Hong Kong, Hong Kong SAR, China; yunlonglm@gmail.com (Y.L.); evywang2017@gmail.com (Y.W.); songzijing@link.cuhk.edu.hk (Z.S.); chloehe@link.cuhk.edu.hk (X.H.); 2 School of Medicine, The Chinese University of Hong Kong-Shenzhen, Shenzhen 518172, China

**Keywords:** pyrrolizidine alkaloids, metabolic activation, genotoxicity, repair pathway

## Abstract

Pyrrolizidine alkaloids (PAs) are common phytotoxins that are found worldwide. Upon hepatic metabolic activation, the reactive PA metabolites covalently bind to DNAs and form DNA adducts, causing mutagenicity and tumorigenicity in the liver. However, the molecular basis of the formation and removal of PA-derived DNA adducts remains largely unexplored. In the present study, Sprague Dawley (SD) rats were exposed to retrorsine (RTS), a representative PA, at a human-relevant dose of 3.3 mg/kg/day for 28 days. The rats were divided into three groups: control, RTS-28 (sacrificed after continuous RTS exposure), and RTS-161 (sacrificed at 133 days post-RTS-exposure). The multi-omics analyses demonstrated the involvement of homologous recombination (HR) and non-homologous end joining (NHEJ) repair pathways as a response to PA-induced DNA damage. Additionally, the characteristic guanine adducts induced by RTS exposure were in accordance with the higher expression of XPA and XPC, indicating that nucleotide excision repair (NER) and base excision repair (BER) also contributed to repairing RTS-induced DNA damage. Furthermore, we also showed that DNA damage persisted after PA exposure, and mutagenically related repair errors might occur due to the prolonged genotoxic effects. The present study lays the foundation for bridging PA-derived DNA adducts, DNA damage, DNA repair, and the follow-up mutagenesis and carcinogenesis associated with PA exposure.

## 1. Introduction

**Figure 1:**
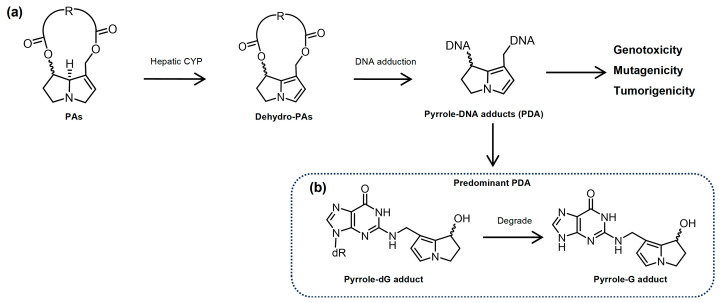
(**a**) Metabolic activation of PAs, the formation of PDA, and resultant genotoxicity, mutagenicity, and tumorigenicity. (**b**) The predominant PDA, pyrrole dG adduct, and its degradation product pyrrole-G adduct.

Pyrrolizidine alkaloids (PAs) are phytotoxins that are present in over 6000 plant species worldwide [1]. Humans are frequently exposed to PAs via the intake of PA-contaminated food products including wheats, pollen, honey, tea, and milk, and such low-level but long-lasting PA exposure might bring about mutagenic and tumorigenic outcomes [2,3,4]. After ingestion, PAs are metabolized by cytochrome P450 enzymes (CYPs) in the liver. The reactive metabolites, namely dehydro-pyrrolizidines (DHPAs), covalently bind to DNA and form pyrrole–DNA adducts (PDAs) (Figure 1) [2,5,6,7]. Although PDAs have been evidenced to cause DNA damage (the initial genotoxic effect of PAs) and liver tumors (the carcinogenic outcome due to PA exposure), the process underlying the PA-induced genotoxicity and mutagenicity, which are the initiating basis of PA-induced tumorigenesis, remains largely unknown.

It has been established that the linkage between genotoxicity and tumorigenicity is the repair of the DNA lesion and the ensued repair errors. For the adducted DNA, the base excision repair (BER) pathway is a specific DNA repair mechanism that targets and repairs damaged or abnormal bases in the DNA molecule [8]. On the other hand, the nucleotide excision repair (NER) pathway is another DNA repair mechanism that focuses on removing various DNA lesions, including bulky adducts, chemical modifications, and UV-induced photoproducts [8,9]. Both pathways play crucial roles in maintaining genomic integrity and preventing the accumulation of mutations caused by base damage. The BER and NER pathways achieve this by excising damaged bases or removing bulky adducts [9,10]. However, whether the BER or NER pathways are involved in repairing PDA, dominated by 7-hydroxy-9-(deoxyguanosin-*N*2-yl) supinidine adducts (pyrrole-G adducts) or 7-hydroxy-9-(deoxyguanosin-*N*2-yl) dehydrosupinidine adducts (pyrrole-dG adduct), remains unknown. Aside from the bulky DNA lesion, DNA double-strand breaks (DSBs) are particularly hazardous [11], and previously, Louisse et al. [11] identified the DSB marker, a phosphorylated H2A histone family member X (γH2AX), in PA-exposed HepaRG cells. The non-homologous end joining (NHEJ) and homologous recombination (HR) repair pathways [12,13] are responsible for repairing DSB. However, it remains unclear whether the NHEJ and HR repair pathways participate in repairing PA-induced DSBs, especially in vivo.

All hepatotumorigenic PAs produce a similar set of DNA adducts in the liver, with pyrrole-dG adducts as the predominant adducts [2]. In 1954, retrorsine (RTS), a representative PA, was shown to induce liver tumors in rats [14]. More recently, studies [15,16] by He et al. have demonstrated that RTS induced liver tumorigenesis in mice, and RTS-exposed HepaRG cells showed signs of mutagenic processes. To add more human relevance, particularly considering the dietary PA exposure pattern, we followed the previous report [17] and established a human-relevant animal model by dosing RTS at a dose of 3.3 mg/kg per day for 28 days. Further, to investigate the DNA repair process after the initial DNA damage induced by RTS exposure, we kept the rats for another 134 days after the end of RTS exposure. By integrating transcriptomic analysis with proteomic studies based on this human-relevant rat model, we explored the DNA repair pathways that are involved in repairing DNA damage induced by PAs, aiming to link the molecular events between PA-induced DNA adducts, DNA lesions, and DNA repair.

## 2. Results

### 2.1. RTS Exposure Upregulates Expression of Genes Related to DNA Damage and Repair, and DNA Repair Pathways Remain Activated Long After Withdrawal of PA Exposure

**Figure 2:**
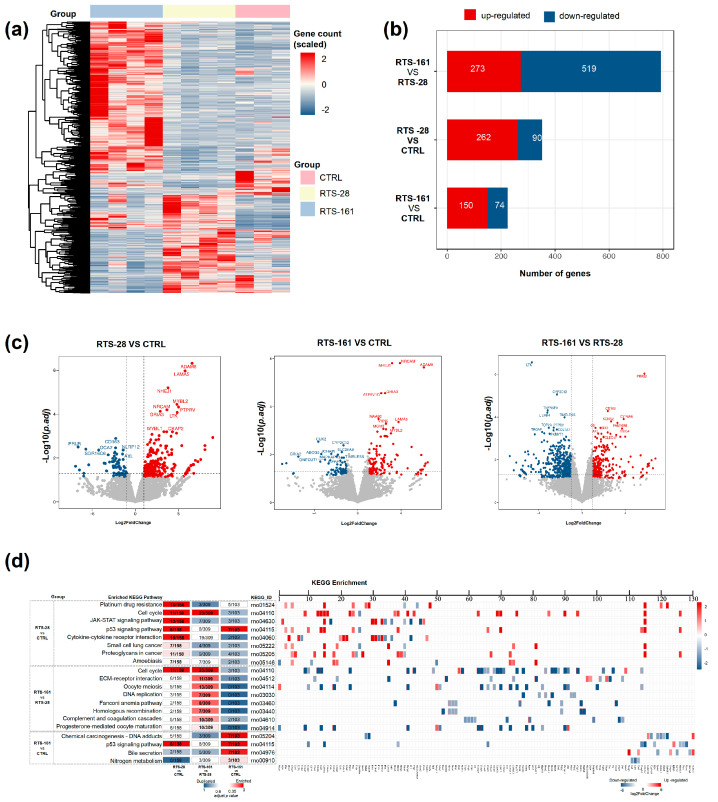
Transcriptomic analyses of rat livers, divided into different groups for comparison. (**a**) Heatmap of DEGs in RTS-28 vs. CTRL, and RTS-161; (**b**) amount of altered gene expressions, divided into different comparison groups: RTS-28 vs. CTRL, RTS-161 vs. CTRL, and RTS-161 vs. RTS-28; (**c**) volcano plot of DEGs for different comparisons; (**d**) KEGG enrichment analysis of DEGs for different comparisons. Data are expressed as mean ± SD (*n* = 4).

Transcriptomic cluster analysis was used to identify DEGs among the CTRL, RTS-161, and RTS-28 groups (Table S1). A heatmap of the transcriptomic analysis for 1065 selected genes in rat livers from these groups was generated (Figure 2a). In order to identify the DEGs across groups and upregulated or downregulated genes for each treatment group, the number of dysregulated genes in the RTS-treated groups were identified, and most of them were upregulated (Figure 2b). Specifically, compared with the CTRL group, 262 genes were upregulated, and 90 genes were downregulated in the RTS-28 group, while 150 genes were upregulated and 74 genes were downregulated in the RTS-161 group. Compared with the RTS-28 group, 519 genes were downregulated and 273 genes were upregulated in the RTS-161 group.

The top 10 differentially expressed genes were indicated in the volcano plot (Figure 2c). Among the differentially expressed genes, non-homologous end joining factor 1 (NHEJ1) encodes a DNA repair factor that is essential for the NHEJ repair pathway, which preferentially mediates repair of DSBs. MYB proto-oncogene like 2 (MYBL2) can encode the protein, which is phosphorylated by cyclin A/cyclin-dependent kinase 2 during the S-phase of the cell cycle. O-6-methylguanine-DNA methyltransferase (Mgmt) can encode the important repair enzyme that is involved in the DNA repair of O(6)-alkylguanine, which is the major mutagenic and carcinogenic lesion in DNA [18]. The increased expressions of NHEJ1, MYBL2, and Mgmt in the RTS-treated groups indicate that RTS provoked DNA damage and induced DNA repair.

To further indicate the genes’ biological functions, we performed a KEGG enrichment analysis of the DEGs (Figure 2d). The significantly overrepresented pathways were, respectively, associated with the cell cycle, JAK-STAT signaling pathway, p53 signaling pathway, and cytokine–cytokine receptor interaction between the CTRL group and RTS-28. A cell cycle, ECM–receptor interaction, DNA replication, Fanconi anemia pathway, homologous recombination, and complement and coagulation cascades were found between the RTS-161 and RTS-28. Chemical carcinogenesis–DNA adducts and a p53 signaling pathway related to tumor initiation were detected between the RTS-161 and CTRL group. The specific genes and the DEG-related pathway are shown in Tables S2–S4.

### 2.2. RTS Exposure Induces Dysregulation of Proteins Linked to DNA Damage Response, Repair Mechanisms, and Xenobiotic Metabolism in Rat Liver

**Figure 3:**
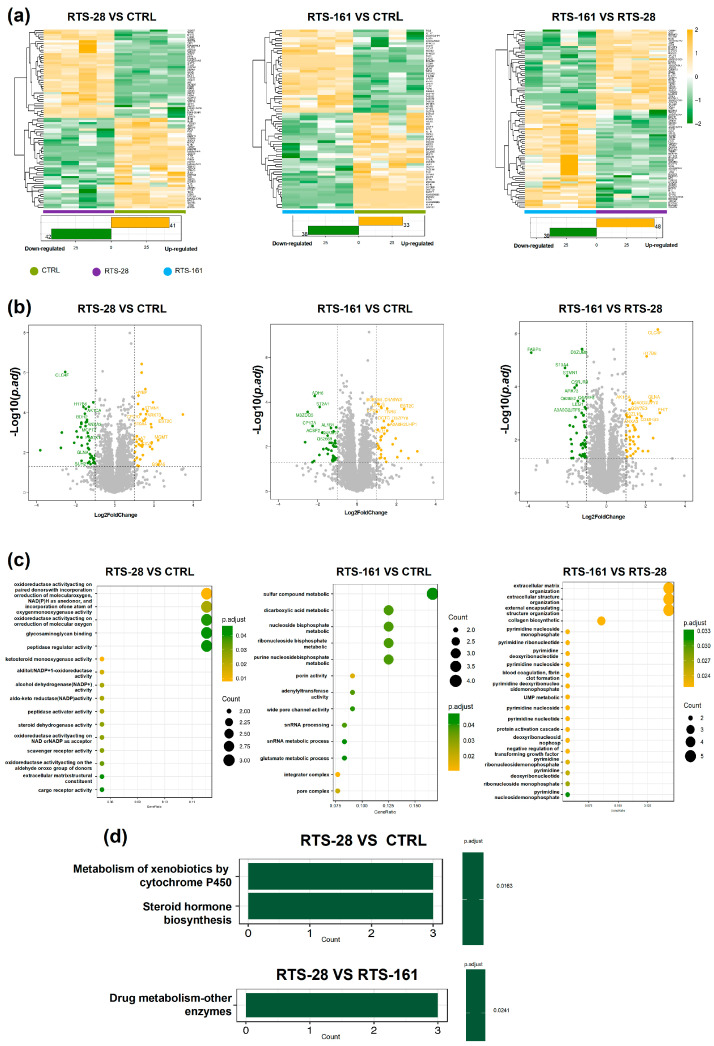
Proteomic analyses of rat livers, divided into different comparison groups. (**a**) Heatmaps of differentially expressed proteins (DEPs), (**b**) volcano plot of DEPs, (**c**) Gene Ontology (GO) annotation classification analysis of DEPs, and (**d**) KEGG enrichment analysis of DEPs. Data are expressed as mean ± SD (*n* = 4).

Meanwhile, proteomic analyses were conducted. The heatmap illustrates the differentially expressed proteins (DEPs) that distinguish the three groups (Figure 3a), indicating that these DEPs may be crucial factors involved in the development of chronic liver injury following RTS administration or during the post-dose period. Volcano plots showed differentially expressed proteins across groups (Figure 3b). Mgmt is an efficient direct DNA repair enzyme that can repair 6-*O*-methylguanine) in the DNA sequence [18]. Mgmt was upregulated in the RTS-28 group. DNA Ligase 1 (LIG1) plays an important role in the BER process and was upregulated by RTS. FHIT is a member of the histidine triad gene family and encodes a diadenosine P1, P3-bis(5′-adenosyl)-triphosphate adenylohydrolase, which is involved in purine metabolism and works as a tumor suppressor. The aberrant expression of Annexin A3 (ANXA3) promotes tumor cell proliferation, invasion, metastasis, and angiogenesis. In order to further investigate the mechanisms of RTS-induced liver injury, GO and KEGG enrichments of proteomics were also performed in rat livers. In the GO results from the proteomics results (Figure 3c), the extracellular matrix organization and collagen biosynthetic process, which are involved in the regulation of cell differentiation processes such as wound repair, showed significant changes in the RTS-28 group, indicating that RTS may lead to fibrosis and damage repair.

In the KEGG analysis of proteomics (Figure 3d), the metabolism of xenobiotics by cytochrome P450, steroid hormone biosynthesis, and the drug metabolism–other enzymes pathways were shown to be highly influenced by RTS.

### 2.3. Multi-Omics Integration Unveils RTS-Induced DNA Damage and Repair Responses in Rat Livers

**Figure 4:**
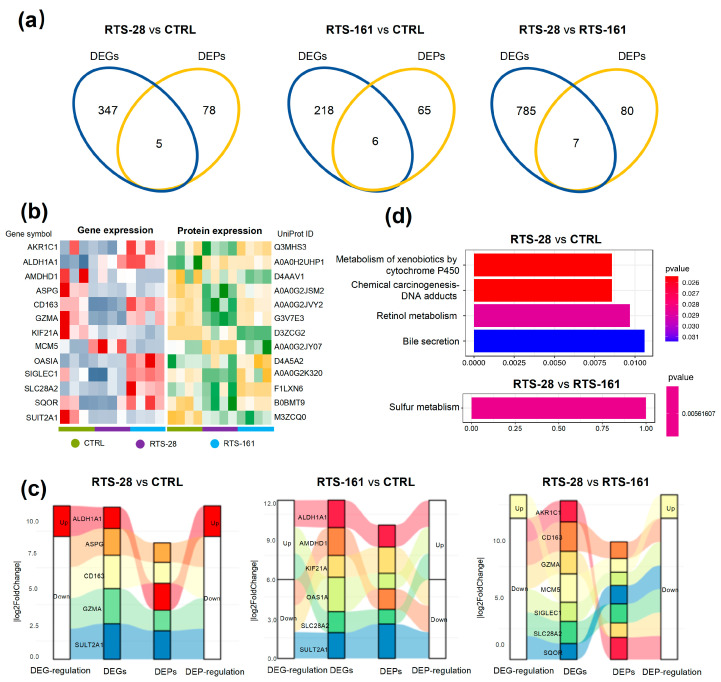
Multi-omics analyses of rat livers, divided into different comparison groups. (**a**) Venn diagram showing overlap among DEPs and DEGs; (**b**) heatmap of correlation between DEGs and DEPs; (**c**) Sankey diagram of correlation between DEGs and DEPs; and (**d**) KEGG pathway analysis of correlation between DEGs and DEPs. Data are expressed as mean ± SD (*n* = 4).

To obtain a comprehensive understanding of RTS-induced DNA damage and DNA repair, the association between transcriptomics and proteomics was further analyzed. A comparative analysis of the transcriptomic and proteomic results between the RTS-treated groups and CTRL groups revealed the presence of coregulated genes, as depicted in Figure 4a. The results showed that five coregulated genes were present between the CTRL group and the RTS-28 group, seven coregulated genes were present between the RTS-161 group and the RTS-28 group, and six coregulated genes were present between the CTRL group and the RTS-161 group (as demonstrated in Figure 4a). The three groups have overlapping genes, which means that there are actually 13 coregulated genes. To effectively analyze these 13 coregulated genes, a heatmap was generated to visualize both the genes and the proteins that they translate. As shown in Figure 4b, rows represent DEGs, and columns represent samples with biological replicates, and the identified DEGs and DEPs were clearly distinguished between the three groups. Thirteen coregulated genes were shown in the heatmaps. In these genes, minichromosome maintenance complex component 5 (MCM5) is predicted to enable single-stranded DNA binding activity and is involved in double-stranded break repair [19]. This component was more upregulated in the RTS-28 group than in the RTS-161 group (Figure 4c), indicating that DNA repair occurred during the post-dose period in rat livers. Tables S5–S7 summarize the scientific information on gene and protein names, changes in gene and protein trends, and other relevant data for each comparable group.

A KEGG pathway analysis was used to identify pathways for these coregulated DEGs and DEPs (Figure 4d). The results showed activation of the cytochrome P450-mediated xenobiotic metabolism, chemical carcinogenesis–DNA adducts, retinol metabolism, and bile secretion pathways in the RTS-28 group compared to the CTRL group, as evidenced by the transcriptomic and proteomic data. The sulfur metabolism pathway was also activated in the RTS-161 group when compared with the RTS-28 group.

Together, the transcriptomic and proteomic data emphasize that RTS-induced liver injury is intricately linked to DNA damage and repair processes. Furthermore, RTS exposure is implicated in promoting mutagenesis and carcinogenesis, alongside the induction of xenobiotic metabolism.

### 2.4. RTS Exposure Induces Chronic Liver Genotoxicity Along with Prolonged DNA Repair, Which May Establish a Basis for Mutagenesis and Carcinogenesis

**Figure 5:**
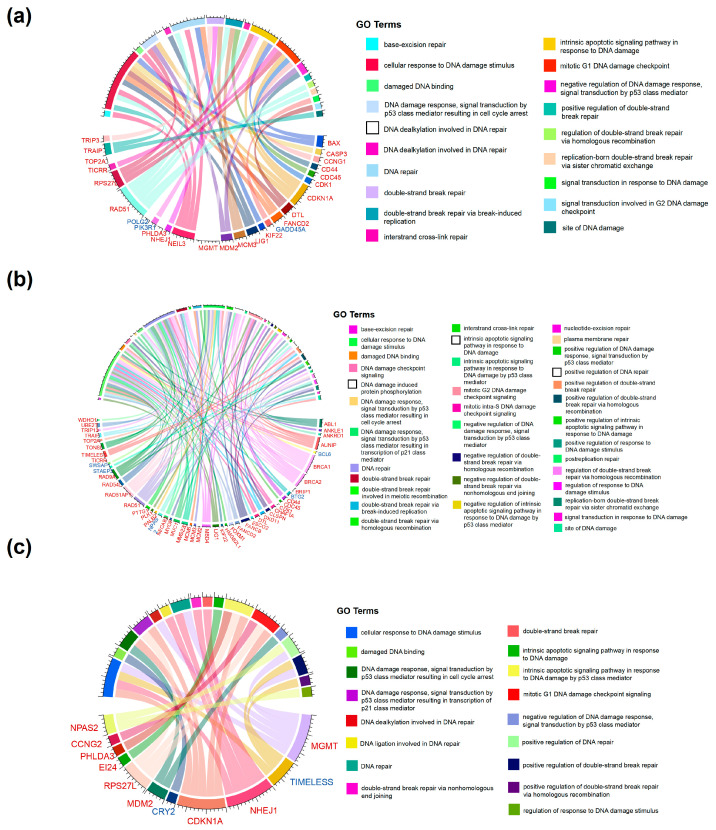
GO annotation classification analysis of DEGs related to DNA damage and DNA repair, divided into different comparison groups: (**a**) RTS-28 vs. CTRL, (**b**) RTS-161 vs. CTRL, and (**c**) RTS-161 vs. RTS-28. Data are expressed as mean ± SD (*n* = 4).

The proteomic and transcriptomic analyses identified a significant number of genes and proteins related to DNA damage and repair. Moreover, several important DNA repair pathways, such as the HR repair pathway, were also detected based on the transcriptomic results, indicating that DNA damage and DNA repair play important roles in RTS-induced chronic liver injury. Therefore, we further performed an in-depth analysis of the transcriptomic data on DNA damage and repair across the three groups (Figure 5a–c). Compared to the CTRL group, dysregulation was observed in several genes associated with DSBs, such as TRIP3, NHEJ1, MCM3, RAD51, CDC45, and MCM3, in the RTS-28 group. Among these genes, RAD51 was found to be associated with the HR repair pathway (Figure 5a), while NEILS was dysregulated and associated with the BER pathway. Additionally, MGMT and LIG were found to be related to DNA dealkylation involved in DNA repair. Furthermore, dysregulation of other genes related to DNA damage was also observed, including TRAIP, NEIL3, BAX, CASP3, CDKN1A, DTL, RPS27L, CDK1, CDKN1A, PIK3R1, GADD45A, and MDM2. NEL3, KIF22, and FANCD22 were also found to be associated with DNA repair.

Compared to the CTRL group, dysregulation was observed in several genes associated with DSBs, including NHEJ1, MGMT, and TIMELESS in the RTS-161 group (Figure 5b). MGMT was found to be associated with DNA dealkylation involved in DNA repair. Dysregulation of other genes related to DNA damage was also observed, including CDKN1A, CRY2, RPS27L, MDM2, NPAS2, EI24, and additional dysregulated genes related to DNA DSBs such as NHEJ1, MGMT, and TIMELESS, which were also associated with DNA repair.

Compared to the RTS-28 group, dysregulation was observed in several genes associated with DNA damage and repair in the RTS-161 group (Figure 5c). Specifically, dysregulation was observed in genes associated with DSBs, such as BRCA1, BRCA2, DDX11, HIMGB211, MCM2, MCM3, MCM5, MYOF, PALB2, PLK, TIMELESS, TONSL, TOP2A, UBE2T, and WDHD1. Moreover, genes related to the HR repair pathway, such as RAD51, RAD51AP1, RAD54L, RAD9A, STAEP3, SWSAP1, and TRIP13, were also found to be dysregulated. Dysregulation of genes related to NER (FANCB and FANCD2), BER (MBD4), and Fanconi anemia (FANCB and FANCD2) was also observed. In addition, other genes related to DNA repair, such as ANKRD1, BRIP1, BTG2, DTL, DSCO2, KIF22, MUC1, NECB3, PTTG1, and NPAS2, were found to be dysregulated, as were genes related to DNA damage, such as AUNIP, CHAF1A, CLSPN, FOXM1, and TICRR.

In summary, the results revealed that RTS-induced liver injury is associated with DNA damage and repair. Notably, DNA repair kept occurring during the post-exposure period: the homologous recombination repair pathway was found to be involved in repairing RTS-induced DNA damage, and dysregulation of genes related to DSBs, single-stranded breaks, NER, BER, and Fanconi anemia was also observed. Our findings suggest that RTS exposure initiated DNA damage, mediated by DNA adducts, followed by DNA repair during the prolonged period after the withdrawal of RTS exposure. The findings provide the basis of DNA repair error and genome alteration, which may further induce mutagenesis and carcinogenesis.

### 2.5. RTS Induced DNA Damage in Rat Livers

**Figure 6:**
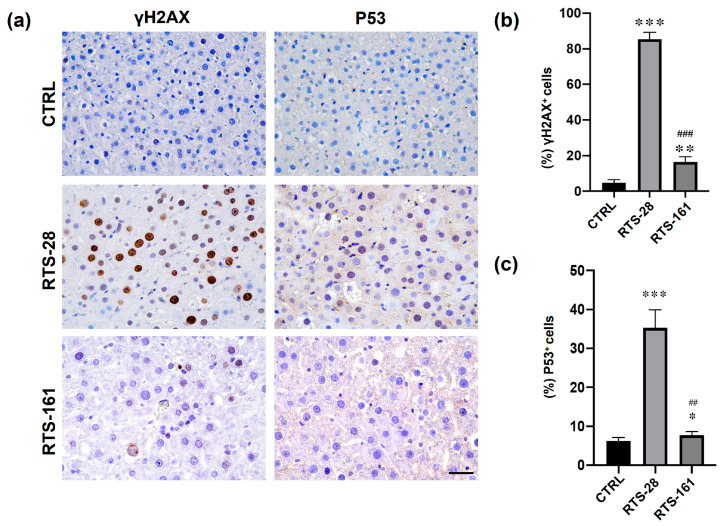
RTS exposure induced DNA damage in rat livers. (**a**) Representative images of immunohistochemistry staining of DNA damage markers (γH2AX and P53). (**b**,**c**) Quantification of γH2AX and P53 expressions in rat livers. * *p* < 0.05, ** *p* < 0.01, *** *p* < 0.001 compared with control group. ^##^ *p* < 0.01, ^###^ *p* < 0.001 compared with RTS-28 group. Data are expressed as mean ± SD (*n* = 3). Scale bars = 50 μm.

Further, we investigated DNA damage and repair manifestations in RTS-exposed rat livers. Immunohistochemistry staining with γH2AX and P53, markers of genotoxicity, showed that both γH2AX and P53 were expressed significantly higher in the RTS-28 group than in the control group, and this effect could be partially restored in the RTS-161 group (Figure 6a–c). These results revealed that 28-day RTS exposure caused obvious DNA damage in rat livers, which could be mostly eliminated by the 134th day after the last RTS dosing.

### 2.6. The BER Pathway and the NER Pathway Were Involved in Repairing RTS-Induced Bulky DNA Lesions in Rat Livers

**Figure 7:**
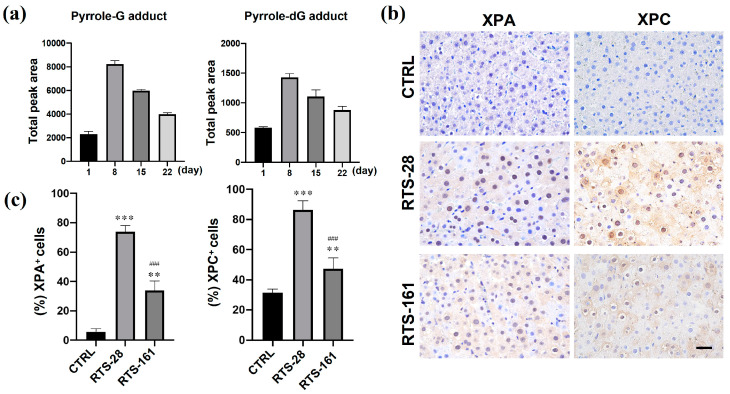
RTS exposure activated the NER and BER pathways in rat livers. (**a**) The urine levels of the pyrrole-G and pyrrole-dG adducts in rat urine detected at different time points during the continuous PA exposure. (**b**) Representative images of the immunohistochemistry staining of NER and BER markers (XPA and XPC). (**c**) Quantification of the expressions of XPA and XPC in rat livers. ** *p* < 0.01, *** *p* < 0.001 compared with the control group, and ^###^ *p* < 0.001 compared with the RTS-28 group. Data are expressed as mean ± SD (*n* = 3). Scale bars = 50 μm.

The BER and NER pathways are capable of repairing DNA damage by eliminating DNA adducts that correspond to specific adducted deoxynucleotides, predominated by the pyrrole-dG adduct, as well as its degraded product, the pyrrole-G adduct. As a result, pyrrole-G adducts serve as biomarkers for BER pathway activation, while pyrrole-dG adducts serve as biomarkers for NER pathway activation. To investigate the role of BER and NER in repairing RTS-induced single-strand breaks, the presence and quantification of pyrrole-dG and pyrrole-G adducts were determined in the urine of RTS-treated rats during the dose and post-dose periods using UHPLC-MS/MS (Figure 7a). The results revealed the presence of pyrrole-G and pyrrole-dG adducts in the urine of rats during the entire dose period. However, no pyrrole-derived adducts were detected in the urine of the control group or the RTS-161 group.

XPA and XPC are two critical factors in the NER pathway and are responsible for recognizing and excising DNA damage in eukaryotic cells [20,21,22]. We compared the expression of these proteins in rat livers. In comparison to control rats, both RTS-28 and RTS-161 rats exhibited significantly increased levels of XPA and XPC in their livers (Figure 7b,c). XPA plays an essential role in the NER pathway by coordinating the assembly of other NER core factors at the site of DNA damage prior to lesion excision [23]. XPC complexes with RAD23B proteins to initiate the repair process [23]. In this study, we measured the protein expressions of XPA and XPC to investigate the involvement of the NER pathway in repairing RTS-induced DNA damage in rat liver. As shown in Figure 7b,c, XPA and XPC proteins were detected in the livers of RTS-treated rats, and their expressions were significantly higher than those of control rats. However, the expression of XPC was decreased in the RTS-161 group compared to the RTS-28 group. These findings indicate the participation of the NER pathway in repairing RTS-induced DNA damage. Although a significant portion of the damage was repaired during the post-dose period, some instances of damage remained unrepaired, which serve as the basis for later mutagenesis.

### 2.7. The HR Repair Pathway and the NHEJ Repair Pathway Were Involved in Repairing RTS-Induced DSB in Rat Livers

**Figure 8:**
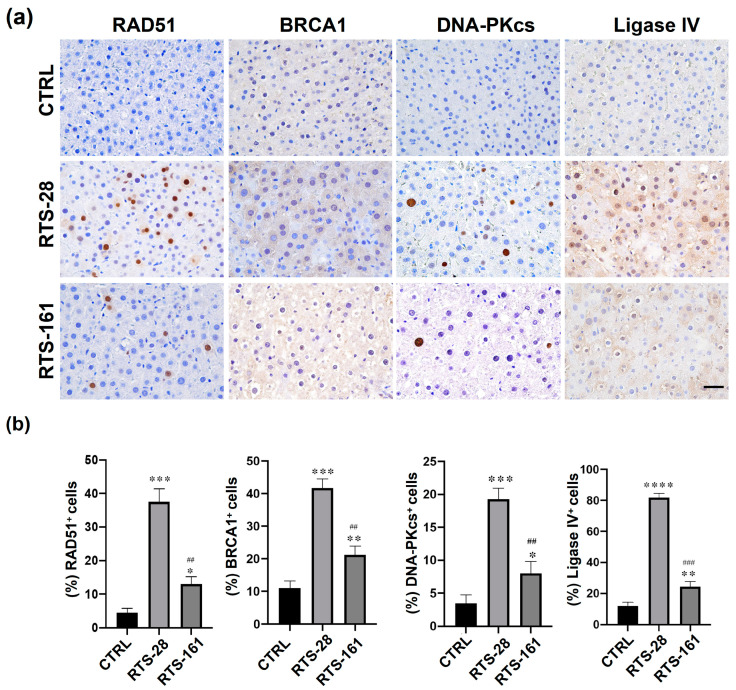
RTS exposure activated HR and NHEJ repair pathways in rat livers. (**a**) Representative images of immunohistochemistry staining of HR and NHEJ markers (RAD51, BRCA1, DNA-PKcs, and Ligase IV). (**b**) Quantification of expressions of RAD51, BRCA1, DNAPK-cs, and Ligase IV in rat livers. * *p* < 0.05, ** *p* < 0.01, *** *p* < 0.001, **** *p* < 0.0001 compared with control group; ^##^ *p* < 0.01, ^###^ *p* < 0.001 compared with RTS-28 group. Data are expressed as mean ± SD (*n* = 3). Scale bars = 50 μm.

HR and NHEJ are essential, prominent mechanisms for DSB repair [24]. In the present study, genes associated with NHEJ and HR were evaluated. To explore the involvement of the HR repair pathway in repairing RTS-induced DNA damage, we carried out immunohistochemistry staining of RAD51 and BRCA1, two important factors in the HR repair pathway, in rat livers (Figure 8a). The results showed that the expressions of RAD51 and BRCA1 were significantly upregulated by RTS treatment compared to the control group, while it was partially blunted in the RTS-161 group (Figure 8a,b). DNA-PKcs is a DNA-dependent protein kinase that is recruited and activated at the site of DNA damage by KU proteins to repair DSBs that are repaired by NHEJ pathways. DNA ligase IV guides the end-processing choice during non-homologous end joining. The immunohistochemistry showed that DNA-PKcs and Ligase IV were expressed significantly higher in the RTS-28 group than in the control group and could be significantly rescued in the RTS-161 group (Figure 8a,b), indicating that the NHEJ repair pathway was activated in the RTS-28 group and the RTS-161 group. These findings revealed that the HR repair pathway and the NHEJ repair pathway were also involved in repairing RTS-induced DSBs in rat livers, and the DNA damage cannot be totally repaired by such mechanisms.

## 3. Discussion

It has been well established that most of the carcinogenesis process derives from mutagenicity, which depends on genotoxicity and whether the DNA damage is fully repaired. It is therefore necessary to investigate the process of PA-induced genotoxicity and DNA repair pathways to reveal the initiating mechanisms and causal link between PA exposure and liver cancer development. Previous reports have demonstrated that different PAs could cause DNA damage at human-relevant concentrations, which are not acutely toxic and relevant to dietary PA exposure [17]. In the present study, we utilized a human-relevant rat model to explore the genotoxicity and mutagenicity induced by PAs. Additionally, we expanded upon previous research by adding a new RTS-161 group to investigate DNA damage and DNA repair pathways after the withdrawal of PA exposure.

In terms of the molecular study of PA-induced genotoxicity, a previous study using whole-genome microarrays [17,25] found that 35 genes were deregulated following PA exposure. Meanwhile, this study utilized transcriptomic analysis, which has a larger dynamic range than microarray assays and is therefore more sensitive when detecting gene expression changes. Consequently, we identified significantly more genes that showed a high degree of overlap with the 35 genes previously identified in the literature, such as Mgmt, MYBL2, and RAD51. This consistency between the present study and previous results strongly supports the idea that a PA-induced DNA repair response underlies its mutagenicity. Moreover, incorporating transcriptomic and proteomic results, the present study identified further information: LIG1 plays an important role in DNA replication, repair, and recombination, and LIG1 defunction has been associated with impaired DNA replication and repair [26]; MDM2 plays a crucial role in regulating the activity of p53, the critical tumor suppressor [27]; and FANCD2 is important for repairing DNA inter-strand crosslinks [28].

For specific genotoxic effects and possible mutagenic event induced by PAs, we looked into the PA-derived chemical carcinogenesis—the formation and repair of DNA adducts. The initial formation of PDA has been evidenced to be the cause of eventual tumors induced by PA exposure. Several case studies report the formation of PDAs in different species, which were further developed as the biomarker of PA-associated tumorigenesis [15], and a previous study in our lab investigated for the first time the dose- and time-dependent accumulation of PDA and the correlation between the levels of DNA adduct formation and the severity of liver damage [5]. To date, most of the reported studies have focused on the formation of PDA [4,29,30], but few studies have unraveled the molecular basis underlying the sequential process from the formation of PDA (DNA bulky lesion) to mutagenesis and, eventually, tumorigenesis [31]. Previous studies have demonstrated the involvement of the BER and NER pathways in repairing DNA adducts in eukaryotic cells [32].

In the present study, PDAs, represented by the pyrrole-dG adduct and pyrrole-G adduct, were detected in the urine of rats. It is worth pointing out that DHP-derived DNA adducts were firstly detected within a non-acute toxic dose range over a 28-day period, reflecting human exposure scenarios through repeated exposure instead of the acute toxic dosage. Our previous study also suggested that solely investigating acute toxicity caused by a single dose may underestimate the consequences of multiple exposures with relatively lower dosages but for prolonged periods, which mimics the PA exposure pattern in humans [5]. With chronic exposure, the accumulation of DNA adducts occurs in a repair-resistant compartment [33]. Comparing the acute exposure, the removal of PDA after continuous exposure was much slower, because many more of the adducts might reside in repair-resistant compartments. Moreover, the presence of the pyrrole-dG adduct and pyrrole-G adduct in rat urine was in accordance with the finding that the NER and BER pathways were involved in repairing PDA.

The BER and NER pathways mainly repair the damage to gene segments caused by DNA adducts [34]. However, when the gene locus or gene segment is not successfully repaired, the damage might develop into more serious DNA damage, such as DSBs. However, few studies have reported the DNA repair pathways that are involved in repairing PA-induced DSBs, which have been revealed to be involved in repairing PA-induced DSBs by our transcriptomic and proteomic results. DSBs can be repaired by NHEJ or HR, also known as recombination repair or template-assisted repair. Our study confirmed the findings based on transcriptomic and proteomic data that RTS treatment activated the HR repair pathway, as evidenced by the involvement of two key factors, such as RAD51 and BRCA1, in this pathway. Additionally, we also found that the NHEJ repair pathway was involved in the repair of RTS-induced DSBs, as indicated by the participation of two important factors, namely DNA-PKcs and DNA Ligase IV enzyme, in this pathway. Furthermore, we found that these repair pathways persisted in post-exposure rat livers, indicating the prolonged genotoxic effect even after the withdrawal of PA exposure, and these events could be the basis for repair errors that might initiate mutagenesis and carcinogenesis.

## 4. Conclusions

Overall, based on a rat model that is relevant to human PA exposure, the present proteomic and transcriptomic study provides preliminary insights into the DNA repair pathways, bridging the initial genotoxic effect, later mutagenic effect, and eventual tumorigenic effect induced by PAs. Our findings provide a foundation for further exploration of the underlying mechanisms of PA-induced carcinogenesis.

## 5. Materials and Methods

### 5.1. Rat Model of PA-Induced DNA Damage

A 28-day oral toxicity study was performed with male rats exposed to RTS (Sigma-Aldrich, St. Louis, MO, USA), a representative PA that is present in various PA-producing plants. A total of 15 male Sprague Dawley (SD) rats (200–220 g) were randomly divided into 3 groups (*n* = 5 rats per group). For the RTS-treated groups, rats (*n* = 10) were orally administered with RTS at a non-acute toxic dose of 3.3 mg/kg per day for 28 days. Half of the rats (*n* = 5/group) were euthanized on the 29th day (initiation stage). The remaining rats (*n* = 5/group) were maintained without further RTS exposure and euthanized on day 162 (advanced stage). For the CTRL groups, a Vehicle compound was administered for 28 days, and the rats were sacrificed on day 29 (Figure S1). Then, all animals were sacrificed, and the liver samples were collected for histopathological and bioinformatics analyses. The 24 h (0–24 h) urine samples of individual rats were collected once a week into sterilized containers kept on ice throughout the study. Collected urine samples were stored at −80 °C until PDA analysis. The volume of individual urine samples was measured and recorded. An aliquot (200 μL) of urine sample was filtered and centrifuged at 20,000× *g* for 15 min at 4 °C. The supernatant was collected, filtered, and subjected to UHPLC–MS/MS analysis. The procedures of the animal experiments were approved by the Animal Experimental Ethics Committee, The Chinese University of Hong Kong, under the regulations of the Hong Kong SAR government.

### 5.2. Immunohistochemical Analysis of Rat Livers

Rat liver sections embedded in paraffin were cut into sections of 5 µm using a rotary microtome with a cooling head (Leica 2235S) and dried at 4 °C overnight. The sections were deparaffinized in xylene and rehydrated using 100%, 95%, 80%, and 70% ethanol solutions sequentially, followed by the incubation in 10 mM sodium citrate buffer at 98 °C for 20 min for antigen retrieval. After blocking with 10% goat serum for 1 h at room temperature, the sections were incubated with primary antibodies against γH2AX (1:200; Thermo Scientific, Carlsbad, CA, USA), RAD51 Recombinase (RAD51) (1:200; Abcam), Breast cancer 1 (BRCA1) (1:200; Santa Cruz), P53 (1:200; Abcam, UK), DNA-dependent protein kinase, catalytic subunit (DNA-PKcs) (1:200; Sigma-Aldrich, Saint Louis, MO, USA), Xeroderma Pigmentosa Group A (XPA) (1:200; Santa Cruz, Santa Cruz, CA, USA), and Xeroderma Pigmentosa Group C (XPC) (1:200; Santa Cruz, Santa Cruz, CA, USA) overnight at 4 °C. Then, the sections were incubated with horseradish peroxidase-conjugated anti-rabbit IgG secondary antibody for 1 h at room temperature. The sections were then incubated with 3,3′-diaminobenzidine for 20 min at room temperature. IHC images were taken using a Q-imaging digital camera under an Axiophot 2 upright microscope.

### 5.3. UHPLC-MS Analysis

The PDA level was determined by UHPLC-MS analysis, performed on an Agilent 6460 Triple Quadrupole LC/MS System using a Waters Acquity BEH C18 column (2.1 × 100 mm, 1.7 mm). The mobile phase of water containing 0.1% formic acid (A) and acetonitrile (B) was used with a gradient elution as follows: 0–5 min, 35–95% B; 5–5.5 min, 95% B; and 5.5–6 min, 95–35% B. The flow rate was 0.3 mL/min. The injection volume was 2 µL. The mass spectrometer was operated in multiple reaction monitoring mode with transition *m*/*z* 287.0 → 136.0 (pyrrole-G adduct) and 403.0 → 269.0 (pyrrole-dG adduct) in positive ion mode with an electrospray ionization interface and mass range of *m*/*z* 100–600. The fragmentor and collision energy were 185 V and 33 V, respectively.

### 5.4. Transcriptomics Analysis

Total RNA was isolated from fresh liver tissue using the E.Z.N.A^®^ Total RNA Kit (OMEGA, Norcross, GA, USA) according to the manufacturer’s instructions. Transcriptomic sequence raw reads were first filtered, and clean reads were obtained after quality control using FastQC and MultiQC. Then, the clean reads were aligned to reference the rat genome by HISAT2. And the Samtools process was conducted to transfer the data format of alignments in order to pass the alignments to StringTie for transcript assembly and quantification. DESeq2 in R was performed to calculate the differentially expressed genes (DEGs) based their feature counts. Genes which had a fold change > 2 and adjusted *p*-value < 0.05 were defined as DEGs.

### 5.5. Proteomics Analysis

Proteins were first extracted from liver tissues in a Lysis buffer with protease and phosphatase inhibitors, using a bead homogenizer, and were quantified by BCA protein assay and qualified using SDS-PAGE. Then, 100 μg of each qualified protein sample was taken to dilute with 50mM NH4HCO3 by 4 times the volume. And then, 2.5 μg of Trypsin enzyme was added, followed by 4 h of incubation at 37 °C. Enzymatic peptides were desalted using a Strata X column and vacuumed to dryness, after which the freeze-dried peptide samples were obtained using the method of high-pH RP separation and reconstituted with mobile phase A (2% ACN, 0.1% FA) and centrifuged at 20,000× *g* for 10 min, and the supernatant was taken for injection. Separation was carried out using a Thermo UltiMate 3000 UHPLC liquid chromatograph. For DIA analysis, LC-separated peptides were ionized by nanoESI and injected to a tandem mass spectrometer Q-Exactive HF X (Thermo Fisher Scientific, San Jose, CA, USA) using DIA (data-independent acquisition) detection mode.

After performing quality control with the mProphet algorithm, the DIA data were analyzed using the iRT peptides for retention time calibration. Then, based on the target–decoy model applicable to SWATH-MS, a false positive control test was performed with FDR 1%, identification and quantification of peptides were carried out, and proteins were obtained. The R package MSstats was used to evaluate significant differences in proteins or peptides across groups based on a fold change >2 and *p* value < 0.05 as the criteria, followed by the functional annotation and enrichment analysis on differential proteins.

### 5.6. Statistical Analysis

Data analyses were performed using GraphPad Prism 8.5 Results were presented as mean ± SD. Statistical analyses were performed using one-way ANOVA and Student’s *t*-test. *p* < 0.05 was considered to be statistically significant. Functional annotations, including Gene Ontology (GO) annotation, KOG annotation, and pathway annotations, were conducted using the AnnotationDbi (v1.54.1) and biomaRt (v2.48.3) in R 4.0.4. GO enrichment and Kyoto Encyclopedia of Genes and Genomes (KEGG) enrichment were performed using the R package cluster Profiler (v4.0.5). Visualization of multi-omics results was conducted with pheatmap (v1.0.12), circlize (v0.4.13), and ggplot2 (v3.3.5) in R 4.0.4.

## Data Availability

The original contributions presented in the study are included in the article/Supplementary Material, further inquiries can be directed to the corresponding authors.

## Supplementary Materials

The following supporting information can be downloaded at: https://www.mdpi.com/article/10.3390/toxins16120538/s1, Table S1: Dosage regimen of rats in different groups; Table S2: Enrichment analysis of the KEGG pathways of DEGs in RTS-161 group; Table S3: Enrichment analysis of the KEGG pathways of DEGs in RTS-28 group; Table S4: Enrichment analysis of the KEGG pathway of DEGs in RTS-161 group compared with RTS-28 group; Table S5: Co-expressed genes and proteins in RTS-28 compared to CTRL Group; Table S6: Co-expressed genes and proteins in RTS-161 group compared to CTRL group; Table S7: Co-expressed genes and proteins in RTS-161 compared to RTS-28 group; Table S8: The list of abbreviations; Figure S1: Dosage regimen of rat experiment; Figure S2: The analysis of pyrrole-G adduct and pyrrole-dG adduct in the urine of rat exposed to RTS. Representative MRM chromatogram of LC–MS/MS analysis of (a) pyrrole-G adduct and (b) pyrrole-dG adduct in rat urine; Figure S3: The process of NER repair pathway involved in repairing PA-induced DNA damage; Figure S4: The process of BER repair pathway involved in repairing PA-induced DNA damage.
