# Supplementary material for: Repair of Retrorsine-Induced DNA Damage in Rat Livers: Insights Gained from Transcriptomic and Proteomic Studies
Source: Toxins (Basel). 2024 Dec 13;16(12):538. doi: 10.3390/toxins16120538 (PMC11679430; doi:10.3390/toxins16120538)
Supplement: Supplementary file 1 [file toxins-16-00538-s001.zip › toxins-3293469-supplementary.pdf]

Supplementary material

Repair of Retrorsine-Induced DNA Damage in Rat Livers: Insights Gained from Transcriptomic and Proteomic Studies

Yun Long, Yiwei Wang, Zijing Song, Xin He, Yisheng He and Ge Lin

Table S1: Dosage regimen of rats in different groups

Group	Treatment	Post-dose
Control	Vehicle for 28 days, sacrificed on day 29	No
RTS-28	RTS (3.3 mg/kg per day for 28 days), sacrificed on day 29	No
RTS-161	RTS (3.3 mg/kg per day for 28 days)	Kept without treatment for 134 days, sacrificed on day 162

Table S2 Enrichment analysis of the KEGG pathways of DEGs in RTS-161 group

Pathway ID	Description	P-value	P-adjust	Gene name
rno01524	Platinum drug resistance	1.898E-06	4.782E-04	BAX, CASP3, GSTA3, GSTM3L, PIK3R1, RGD1307603, TOP2A, CDKN1A, GSTA3, GSTM3L, CDKN1A, FAS
rno04110	Cell cycle	1.496E-05	1.885E-03	MDM2, CCNA2, CDC45, ESPL1, PKMYT1, PTTG1 CCNA2, CDC45, CDK1, CDKN1C, FAS, GADD45A, MCM3
rno04630	JAK-STAT signaling pathway	2.313E-05	1.943E-03	CDKN1A, AOX1, CISH, PRLR, IFNLR1, IL13RA2, CISH, SOCS2, IL6ST, AOX1, PIK3R1, IL22RA1, LEPR, STAT6
rno04115	p53 signaling pathway	4.225E-05	2.662E-03	CDKN1A, CDK1, CCNG1, GADD45A, MDM2, CASP3, FAS, BAX
rno04060	Cytokine-cytokine receptor	8.453E-05	4.260E-03	BMP3, CCL19, CCL24, CSF1, CX3CR1, EDA2R, IFNLR1, IL13RA2, IL1RN, IL22RA1, IL6ST, LEPR, PRLR, TNFSF13
rno05205	Proteoglycans in cancer	1.137E-03	4.381E-02	MDM2, CDKN1A, COL1A1, CD44, MMP2, PIK3R1, GPC3, FZD4, FAS, CD44, CASP3, CAV1
rno05222	Small cell lung cancer	1.217E-03	4.381E-02	CDKN1A, LAMA5, COL4A1, PIK3R1, LAMA5, GADD45A, COL4A1, CDKN1A, CASP3, BAX
rno05146	Amoebiasis	1.561E-03	4.917E-02	ADCY1, LAMA5, COL4A1, COL1A1, ADCY1, CASP3

Table S3 Enrichment analysis of the KEGG pathways of DEGs in RTS-28 group

Pathway ID	Description	P-value	P-adjust	Gene name
rno04115	p53 signaling pathway	1.995E-05	2.497E-03	IGFBP3, EI24, CDKN1A, CCNG1, CCNE1, MDM2, COL1A1, RPRM, PERP, MDM2
rno05204	Chemical carcinogenesis-DNA adducts	2.601E-05	2.497E-03	SULT2A1, GSTM3L, GSTA6, GSTA3, EPHX1, CYP2C22, CYP2C12, GSTM3L, GSTA3
rno04976	Bile secretion	1.027E-04	6.575E-03	HMGCR, EPHX1, CAR2, ABCG5, SULT2A1, SLCO1A4, KCNN2
rno00910	Nitrogen metabolism	9.142E-04	4.388E-02	CA8, CAR1, CAR2

Table S4 Enrichment analysis of the KEGG pathway of DEGs in RTS-161 group compared with RTS-28 group

Pathway ID	Description	P-value	P-adjust	Gene name
rno04110	Cell cycle	5.357E-11	1.511E-08	CCNA2, CDC45, CDK1, COL1A1, CDKN1C, ESPL1, FAS, GADD45A, MCM3, MDM2, PKMYT1, PTTG1, CCNA2, CCNA2, CDK1, ESPL1, MCM3, CDKN1A
rno03030	DNA replication	1.669E-04	1.839E-02	FANCD2, LIG1, MCM2, MCM5, POLA2, POLE2
rno04512	ECM-receptor interaction	1.994E-04	1.839E-02	VWF, TNC, THBS4, SV2A, SDC4, RGD1565355, LAMA5, COL4A1, CD44, BDKRB2, LAMA5, COL4A1
rno04114	Oocyte meiosis	2.609E-04	1.839E-02	ADCY1, CCNG1, CDK1, COL1A1, ESPL1, PKMYT1, ADCY7, VWF, VSIG4, SERPINE1, ITGAX, CFD, C6, C1QC, C1QB, C1QA, CCNE1, CDC20, HMMR, MAPK11, PLK1, SGO1
rno03460	Fanconi anemia pathway	3.376E-04	1.904E-02	UBE2T, RAD51, PALB2, MCM3, FANCB, BRIP1, BRCA2, BRCA1, MCM3
rno03440	Homologous recombination	4.669E-04	2.194E-02	RAD54L, RAD51, PALB2, BRIP1, BRCA1, BARD1
rno04610	Complement and coagulation cascades	7.610E-04	3.065E-02	VWF, VSIG4, SERPINE1, ITGAX, CFD, C6, C1QC, C1QB, C1QA
rno04914	Progesterone-mediated oocyte maturation	1.290E-04	4.547E-02	PLK1, PKMYT1, MAPK11, KIF22, HMMR, COL1A1, CDK1, CCNB1, CCNA2, ADCY7, ADCY1, ADCY1

Table S5 Co-expressed genes and proteins in RTS-28 compared to CTRL Group

	Gene name	Uniprot ID	Log2 Foldchange	P-value	Gene and protein changes
1	ALDH1A1	A0A0H2UHP1	1.447	1.610E-04	Up
2	ASPG	A0A0G2JSM2	-1.845	9.452E-07	Down
3	CD163	A0A0G2JVY2	-2.268	4.394E-12	Down
4	GZMA	G3V7E3	-2.412	3.271E-08	Down
5	SULT2A1	M3ZCQ0	2.432	1.663E-07	Up

Table S6 Co-expressed genes and proteins in RTS-161 group compared to

	Gene name	Uniprot ID	Log2 Foldchange	P-value	Gene and protein changes
1	ALDH1A1	A0A0H2UHP1	1.992	2.442E-07	Up
2	AMDHD1	D4AAV1	-2.042	9.026E-04	Down
3	KIF21A	D3ZCG2	-1.568	2.205E-05	Down
4	OAS1A	D4A5A2	2.514	2.969E-07	Up
5	SLC28A2	F1LXN6	1.527	1.092E-05	Up
6	SULT2A1	M3ZCQ0	-1.884	1.840E-05	Down

Table S7 Co-expressed genes and proteins in RTS-161 compared to RTS-28 group

Number	Gene name	Uniprot ID	Log2 Foldchange	P-value	Gene and protein changes
1	AKR1C1	Q3MHS3	1.709	8.263E-06	Up
2	CD163	A0A0G2JVY2	2.380	1.588E-22	Up
3	GZMA	G3V7E3	1.812	5.347E-08	Up
4	MCM5	A0A0G2JY07	-2.286	8.274E-09	Down
5	SIGLEC1	A0A0G2K320	1.545	3.614E-05	Up
6	SLC28A2	F1LXN6	1.799	6.982E-10	Up
7	SQOR	B0BMT9	1.288	3.042E-05	Up

Table S8 The list of abbreviations

ID	Abbr.	Full Name	ID	Abbr.	Full Name
**1**	CYP	Cytochromes P450	**31**	*Cd44*	CD44 Molecule
**2**	PAs	Pyrrolizidine alkaloids	**32**	*Cdc20*	Cell Division Cycle 20
**3**	PDA	Pyrrole-DNA adduct	**33**	*Cdc45*	Cell Division Cycle 45
**4**	PPA	Pyrrole-protein adduct	**34**	*Cdk1*	Cyclin Dependent Kinase 1
**5**	DSBs	DNA double-strand breaks	**35**	*Cdkn2c*	Cyclin Dependent Kinase Inhibitor 2C
**6**	γH2AX	Phosphorylated H2A histone	**36**	*Cfd*	Complement Factor D
**7**	DEPs	Differentially expressed proteins	**37**	*Col1a1*	Collagen Type I Alpha 1 Chain
**8**	pyrrole–dG adducts	7-hydroxy-9-(deoxyguanosin-N2-yl) dehydrosupinidine adducts	**38**	*Col4a1*	Collagen Type IV Alpha 1 Chain
**9**	pyrrole–G adducts	7-hydroxy-9-(deoxyadenosin-N6-yl) supinidine adducts	**39**	*Espl1*	Extra Spindle Pole Bodies Like 1
**10**	GO	Gene Ontology	**40**	*Fancb*	FA Complementation Group B
**11**	KEGG	Kyoto Encyclopedia of Genes and Genomes	**41**	*Fancd2*	FA Complementation Group D2
**12**	NER	Nucleotide excision repair	**42**	*Hmmr*	Hyaluronan Mediated Motility Receptor
**13**	BER	Base excision repair	**43**	*Itgax*	Integrin Subunit Alpha X
**14**	HR	Homologous recombination	**44**	*Kif22*	Kinesin Family Member 22
**15**	NHEJ	Non-homologous end joining	**45**	*Lama5*	Laminin Subunit Alpha 5
**16**	RAD51	RAD51 Recombinase	**46**	*Lig1*	DNA Ligase 1
**17**	BRCA1	Breast cancer 1	**47**	*Mapk11*	Mitogen-Activated Protein Kinase 11
**18**	DNA-PKcs	DNA-dependent protein kinase	**48**	*Mcm2*	Minichromosome Maintenance Complex
**19**	XPA	Xeroderma Pigmentosa Group A	**49**	*Mcm3*	Minichromosome Maintenance Complex
**20**	XPC	Xeroderma Pigmentosa Group A	**50**	*Mcm5*	Minichromosome Maintenance Complex
**21**	*Ephx1*	Epoxide Hydrolase 1	**51**	*Mcm6*	Minichromosome Maintenance Complex
**22**	*Gsta3*	Glutathione S-Transferase Alpha	**52**	*Orc6*	Origin Recognition Complex Subunit 6
**23**	*Gsta6*	Glutathione S-Transferase Alpha	**53**	*Palb2*	Partner And Localizer Of BRCA2
**24**	*Gstm3l*	Glutathione S-Transferase Mu 31	**54**	*Pkmyt1*	Protein Kinase, Membrane Associated
**25**	*Hmgcr*	3-Hydroxy-3-Methylglutaryl-CoA	**55**	*Plk1*	Polo Like Kinase 1
**26**	*Igfbp3*	Insulin Like Growth Factor	**56**	*Pola2*	DNA Polymerase Alpha 2, Accessory Subunit
**27**	*Kcnn2*	Potassium Calcium-Activated	**57**	*Pole2*	DNA Polymerase Epsilon 2, Accessory
**28**	*Mdm2*	MDM2 Proto-Oncogene	**58**	*Pttg1*	PTTG1 Regulator of Sister Chromatid
**29**	*Perp*	P53 Apoptosis Effector Related	**59**	*Rad51*	RAD51 Recombinase
**30**	*Rprm*	Reprimo	**60**	*Rad54l*	RAD54 Like

**61**	*Slco1a4*	Solute Carrier Organic Anion	**96**	*RGD156*	CD36
**62**	*Sult2a1*	Sulfotransferase Family 2A	**97**	*Sdc4*	Syndecan 4
**63**	*Il22ra1*	Interleukin 22 Receptor Subunit	**98**	*Serpine1*	Serpin Family E Member 1
**64**	*Il6st*	Interleukin 6 Cytokine Family	**99**	*Sgo1*	Shugoshin 1
**65**	*Adcy1*	Adenylate Cyclase	**100**	*Cdkn1a*	Cyclin Dependent Kinase Inhibitor 1A
**66**	*Aox1*	Aldehyde Oxidase 1	**101**	*Cdkn1c*	Cyclin Dependent Kinase Inhibitor 1C
**67**	*Bax*	BCL2 Associated X	**102**	*Cish*	Cytokine Inducible SH2 Containing Protein
**68**	*Bmp3*	Bone Morphogenetic Protein 3	**103**	*Col1a1*	Collagen Type I Alpha 1 Chain
**69**	*Casp3*	Caspase 3	**104**	*Col4a1*	Collagen Type IV Alpha 1 Chain
**70**	*Cav1*	Caveolin 1	**105**	*Csf1*	Colony Stimulating Factor 1
**71**	*Ccl19*	C-C Motif Chemokine Ligand 19	**106**	*Cx3cr1*	C-X3-C Motif Chemokine Receptor 1
**72**	*Ccl24*	C-C Motif Chemokine Ligand 24	**107**	*Eda2r*	Ectodysplasin A2 Receptor
**73**	*Ccna2*	Cyclin A2	**108**	*Espl1*	Extra Spindle Pole Bodies Like 1
**74**	*Ccng1*	Cyclin G1	**109**	*Fas*	Fas Cell Surface Death Receptor
**75**	*Cd14*	CD14 Molecule	**110**	*Fzd4*	Frizzled Class Receptor 4
**76**	*Cd44*	CD44 Molecule	**111**	*Gadd45a*	Growth Arrest and DNA Damage Inducible
**77**	*Cdc45*	Cell Division Cycle 45	**112**	*Gpc3*	Glypican 3
**78**	*Cdk1*	Cyclin Dependent Kinase 1	**113**	*Gsta3*	Glutathione S-Transferase Alpha 3
**79**	*Gstm3l*	Glutathione S-Transferase Mu 3	**114**	*Ifnlr1*	Interferon Lambda Receptor 1
**80**	*Lama5*	Laminin Subunit Alpha 5	**115**	*Sv2a*	Synaptic Vesicle Glycoprotein 2A
**81**	*Lepr*	Leptin Receptor	**116**	*Tgfb2*	
**828**	*Mcm3*	Minichromosome Maintenance	**117**	*Thbs4*	Thrombospondin 4
**83**	*Mdm2*	MDM2 Proto-Oncogene	**118**	*Tnc*	Tenascin C
**84**	*Mmp2*	Matrix Metallopeptidase 2	**119**	*Ttk*	TTK Protein Kinase
**85**	*Pik3r1*	Phosphoinositide-3-Kinase	**120**	*Ube2t*	Ubiquitin Conjugating Enzyme E2 T
**86**	*Pkmyt1*	Protein Kinase, Membrane	**121**	*Vsig4*	V-Set And Immunoglobulin Domain
**87**	*Prlr*	Prolactin Receptor	**122**	*Vwf*	Von Willebrand Factor
**88**	*Pttg1*	PTTG1 Regulator Of Sister	**123**	*Wee1*	WEE1 G2 Checkpoint Kinase
**89**	*RGD13076*	No name	**124**	*Abcg5*	ATP Binding Cassette Subfamily G Member
**90**	*Socs2*	Suppressor Of Cytokine Signaling	**125**	*Ca8*	Carbonic Anhydrase 8
**91**	*Stat6*	Signal Transducer And Activator	**126**	*Car1*	Carbonic Anhydrase 1
**92**	*Tnfsf13*	TNF Superfamily Member 13	**127**	*Car2*	Carbonic Anhydrase 2
**93**	*Top2a*	DNA Topoisomerase II Alpha	**128**	*Ccng1*	Cyclin G1
**94**	*Abl1*	ABL Proto-Oncogene 1	**129**	*Cdkn1a*	Cyclin Dependent Kinase Inhibitor 1A
**95**	*Adcy1*	Adenylate Cyclase 1	**130**	*Cyp2c12*	Cytochrome P450 Family 2 Subfamily C

**131**	*Adcy7*	Adenylate Cyclase 7	**147**	*Cyp2c22*	Cytochrome P450 Family 2 Subfamily C
**132**	*Bard1*	BRCA1 Associated RING Domain	**148**	*Ei24*	EI24 Autophagy Associated Transmembrane
**133**	*Bdkrb2*	Bradykinin Receptor B2	**149**	*Ephx1*	Epoxide Hydrolase 1
**134**	*Brca1*	BRCA1 DNA Repair Associated	**150**	*Gsta3*	Glutathione S-Transferase Alpha 3
**135**	*Brca2*	BRCA2 DNA Repair Associated	**151**	*Gsta6*	Glutathione S-Transferase Alpha 6
**136**	*Brip1*	BRCA1 Interacting Helicase 1	**152**	*Gstm3l*	Glutathione S-Transferase Mu 31
**137**	*Bub1*	BUB1 Mitotic Checkpoint	**153**	*Hmgcr*	3-Hydroxy-3-Methylglutaryl-CoA Reductase
**138**	*Bub1b*	BUB1 Mitotic Checkpoint	**154**	*Igfbp3*	Insulin Like Growth Factor Binding Protein 3
**139**	*C1qa*	Complement C1q A Chain	**155**	*Kcnn2*	Potassium Calcium-Activated Channel
**140**	*C1qb*	Complement C1q B Chain	**156**	*Mdm2*	MDM2 Proto-Oncogene
**14**	*C1qc*	Complement C1q C Chain	**157**	*Perp*	P53 Apoptosis Effector Related To PMP22
**142**	*C6*	Complement C6	**158**	*Rprm*	Reprimo
**143**	*Ccna2*	Cyclin A2	**159**	*Slco1a4*	Solute Carrier Organic Anion Transporter
**144**	*Ccnb1*	Cyclin B1	**160**	*Sult2a1*	Sulfotransferase Family 2A Member 1
**145**	*Ccnd2*	Cyclin D2	**159**		
**146**	*Ccne1*	Cyclin E1	**160**		

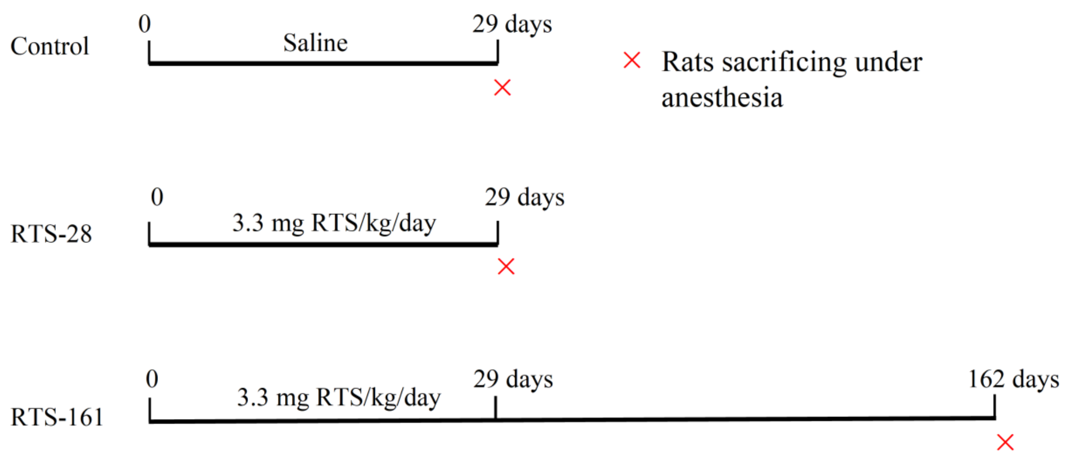

Figure S1: Dosage regimen of rat experiment.

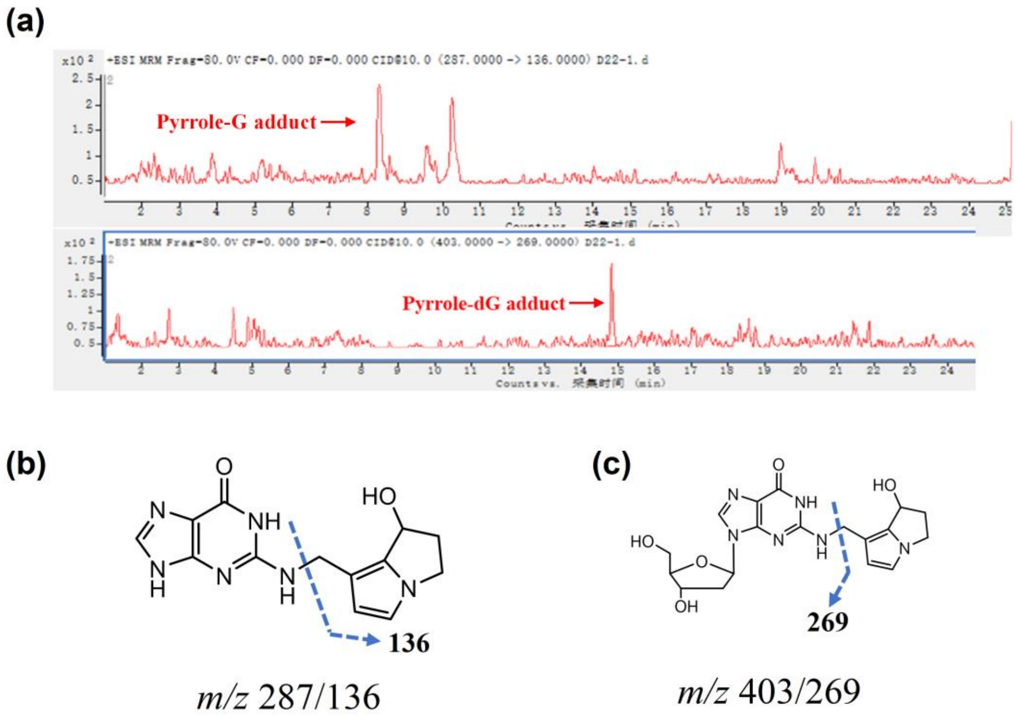

Figure S2: The analysis of pyrrole-G adduct and pyrrole-dG adduct in the urine of rat exposed to RTS. Representative MRM chromatogram of LC–MS/MS analysis of (a) pyrrole-G adduct and (b) pyrrole-dG adduct in rat urine.

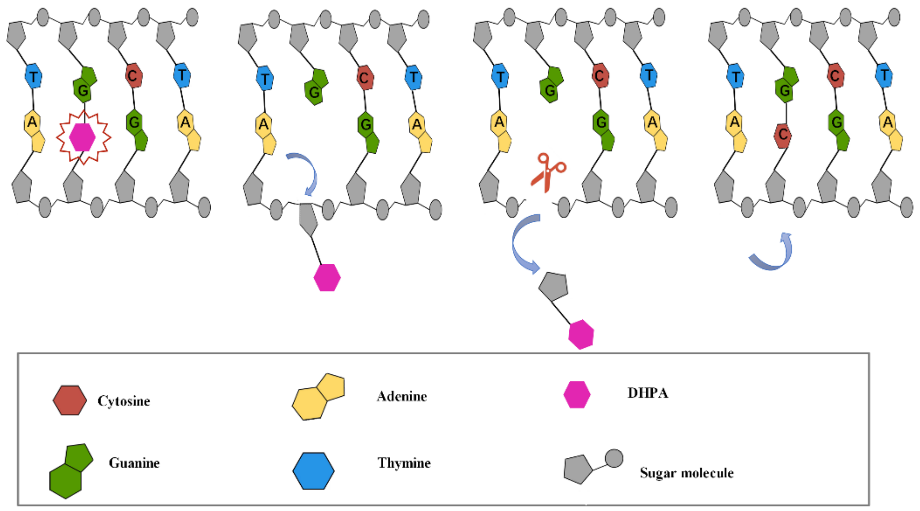

Figure S3: The process of NER repair pathway involved in repairing PA-induced DNA damage.

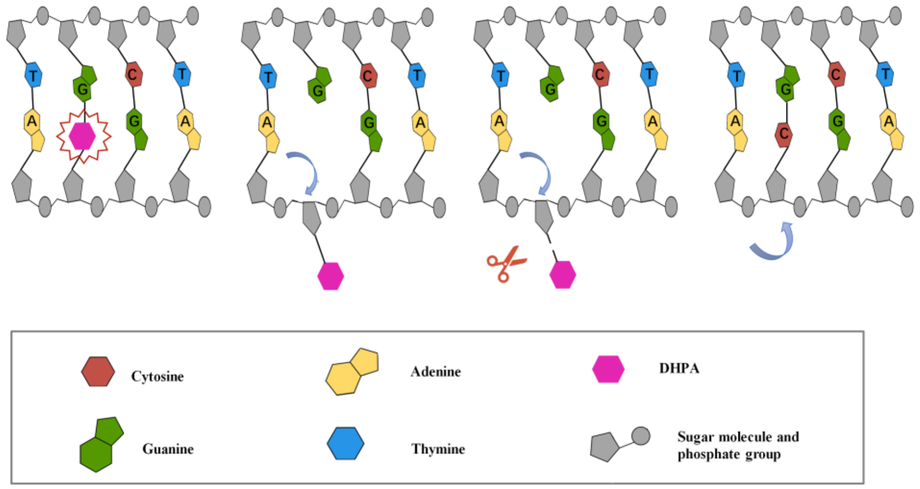

Figure S4: The process of BER repair pathway involved in repairing PA-induced DNA damage.
